# Prevalence and Infection Intensity of Human and Animal Tungiasis in Napak District, Karamoja, Northeastern Uganda

**DOI:** 10.3390/tropicalmed8020111

**Published:** 2023-02-11

**Authors:** Francis Mutebi, Hannah McNeilly, Marlene Thielecke, Felix Reichert, Susanne Wiese, George Mukone, Hermann Feldmeier

**Affiliations:** 1 School of Veterinary Medicine and Animal Resources, College of Veterinary Medicine, Animal Resources and Biosecurity, Makerere University, Kampala P.O. Box 7062, Uganda; 2 Innovations for Tropical Disease Elimination (IFOTRODE), Kampala P.O. Box 24461, Uganda; 3 Edinburgh Medical School: Biomedical Sciences, The University of Edinburgh, Edinburgh EH8 9XD, UK; 4 Charité Center for Global Health, Institute of International Health, Charité-University Medicine Berlin, Corporate Member of Freie Universität Berlin and Humboldt-Universität zu Berlin, 10117 Berlin, Germany; 5 Department of Infectious Disease Epidemiology, Robert Koch Institute, 13353 Berlin, Germany; 6 Institute of Microbiology, Infectious Diseases and Immunology, Charité-University Medicine Berlin, Corporate Member of Freie Universität Berlin, Humboldt-Universität zu Berlin, and Berlin Institute of Health, 10117 Berlin, Germany

**Keywords:** tungiasis, epidemiology, control, population, Uganda

## Abstract

Tungiasis is an important but highly neglected cause of morbidity in resource-poor communities in Latin America and sub-Saharan Africa. Data upon which implementation of control measures can be based are scarce. Before piloting an integrated tungiasis control program in three parishes of Napak district, Uganda, a cross-sectional survey involving the systematic examination of humans and domestic mammals was implemented to establish the occurrence patterns of tungiasis. The study population was 5482 residents, of which 4035 (73.6%) participated in the study. The prevalence of tungiasis in humans was 62.8% (95% CI: 61.3–64.3%), with slightly more males than females affected (*p* = 0.01). Age-specific prevalence and intensity of human tungiasis followed an S-curve pattern, with children of 5–14 years and the elderly (≥60 years) being the most affected. Half of all lesions (50%) had been manipulated by sharp objects. The prevalence of tungiasis in animals was lower (14.2%, 95% CI: 10.9–18.0) than that of humans (*p* < 0.001). Animal tungiasis occurred in decreasing order of frequency in pigs (80%), dogs (24%), goats (16.3%), cats (8.1%) and sheep (4.9%). In conclusion, human tungiasis was highly prevalent but animal infections were comparatively few in the study area. Nevertheless, effective control measures should be based on One Health principles.

## 1. Introduction

Tungiasis (sand flea disease) is an important cause of morbidity among humans and animals in many resource-poor communities in South America, the Caribbean and sub-Saharan Africa [1]. Among humans, children of school age, the elderly, the disabled and drug addicts are among the most vulnerable groups in the population [2]. Moreover, the intensity of sand flea disease is highly variable among affected individuals [3]. While most patients carry only a few sand fleas, a minority carry most of the lesions [4,5,6]. Very severe cases of human and animal tungiasis, with hundreds and sometimes thousands of penetrated sand fleas, have been described in endemic areas [7,8,9]. Tungiasis has also been described outside endemic areas among immigrants [10] and returning tourists [11]. While immigrants and residents in endemic areas may present with severe infections, tourists usually present with one or two lesions [10,11]. 

Among the 14 species of sand fleas [12], only *Tunga penetrans* has been reported to occur in Africa. The parasite affects a wide range of mammalian hosts [13,14,15]. Apart from humans, various wild and domestic animals, including pigs, dogs, cats, small ruminants and peri-domestic rodents, are hosts for *T. penetrans* [15,16,17,18]. Within endemic communities, tungiasis has a heterogeneous distribution. The life cycle of sand fleas is completed in two phases: an environmental development phase and an on-host development phase. Off-host development requires dry weather conditions and dust for the eggs to develop into larvae, pupae, and adults, as is the case with other species of fleas [19,20]. Upon emergence from cocoons, both male and female sand fleas actively search for suitable hosts to feed (haematophagy) [19], but only the female fleas permanently embed in the skin. Embedded fleas remain in contact with the environment through their last abdominal segments, through which they are fertilised, excrete faeces, breath and expel eggs. While embedded in the skin, female sand fleas undergo neosomic development in about two weeks and reproduce before they die in situ after 4–6 weeks from the time of penetration [19,21]. 

Tungiasis is estimated to occur in 89 countries in Latin America and sub-Saharan Africa [22,23]. However, epidemiological studies remain limited, and in many endemic communities, tungiasis occurrence patterns have not been described. Available studies indicate high variability in the epidemiological patterns of tungiasis among endemic areas and study subjects. Community-based studies in Brazil have reported human tungiasis prevalences of up to 54.8% [16,18,24,25,26]. In sub-Saharan Africa, studies from Kenya, Cameroon, Ethiopia and Nigeria have reported a high prevalence of human tungiasis of up to 62.1% [6,27,28,29,30,31], 53% [32,33], 58.7% [34,35] and 42.5% [5,36,37], respectively. In Rwanda, Tanzania and Uganda, studies have reported prevalences of up to 23% [38], 42.5% [39] and 22.5% [40], respectively. In most studies, the prevalence and intensity of human tungiasis infections are higher among males than females [6,29,41,42]. These differences have been attributed to variations in behaviour and hygienic practices. However, a few studies report no significant differences in occurrence patterns between males and females [5,24,34].

The role of animal reservoirs in the transmission cycles of *T. penetrans* differs among endemic communities; while pigs are the key animal hosts of sand fleas in sub-Saharan Africa, with a prevalence of up to 54.8% [15,17], in Brazil, dogs, cats and peri-domestic rodents are considered the most important hosts [16,18,43]. In Brazil, high prevalences were recorded in dogs (67.1%), cats (49.6%) and rats (41.2%) [16,18,25]. Animal infections increase the occurrence of off-host stages of *T. penetrans*, thereby increasing the risk of tungiasis in humans [14,18,44,45].

Penetration of sand fleas in the skin of the host evokes an inflammatory response that is characterised by intense pain and pruritus [46]. Severe tungiasis-associated morbidity ensues from repeated flea penetration and the build-up of high numbers of viable sand fleas in the skin [7,47]. Secondary bacterial infections often exacerbate morbidity [48,49,50,51]. Affected persons and animals present with various clinical signs or lesions, including tissue necrosis, deep ulcers, abscesses, phlegmon, osteomyelitis, anaemia, gangrene and feet or toe amputations [5,7,46]. Some manifestations of tungiasis are life-threatening [52,53], while others may result in permanent physical deformation and disability [54,55]. The characteristic skin lesions of tungiasis are conspicuous and easily detectable. Thus, at the community level, tungiasis may contribute to stigma and social exclusion [56] and poor school attendance [38] and performance among school-going children. Reduced mobility among adults may lower their economic productivity. Thus, tungiasis is an important driver of poverty among affected households and communities [57]. 

In Uganda, tungiasis has been prioritised for control as a neglected tropical disease (NTD) by the Ministry of Health (MOH) [58]. Nevertheless, to date, neither active nor passive surveillance programs for tungiasis are in place. Thus, little data are available on the epidemiology of tungiasis, and an effective control strategy has not been developed. To date, the most widely applied tungiasis treatment method in endemic communities is the extraction of sand fleas with non-sterilised sharp instruments [59]. This method is very painful, destroys normal tissues and poses a risk of secondary bacterial infections, including tetanus [53]. 

The objectives of the present study are: (1) an assessment of the prevalence and intensity of *T. penetrans* infections among humans and household-associated animals in Karamoja, Northeastern Uganda, and (2) to determine the proportion of manipulated sand flea lesions to understand the extent of the problem of manual extraction methods. 

## 2. Materials and Methods

### 2.1. Study Area and Study Population

**Figure 1:**
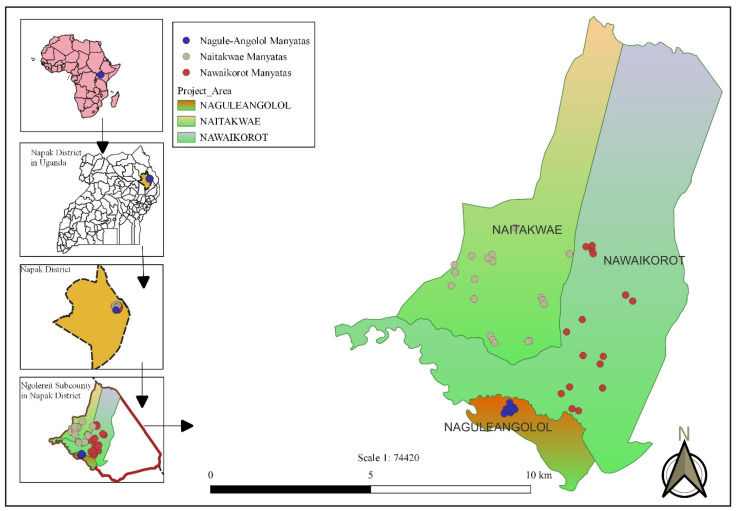
Map showing the location of the *manyatas* in the three study parishes.

**Figure 2:**
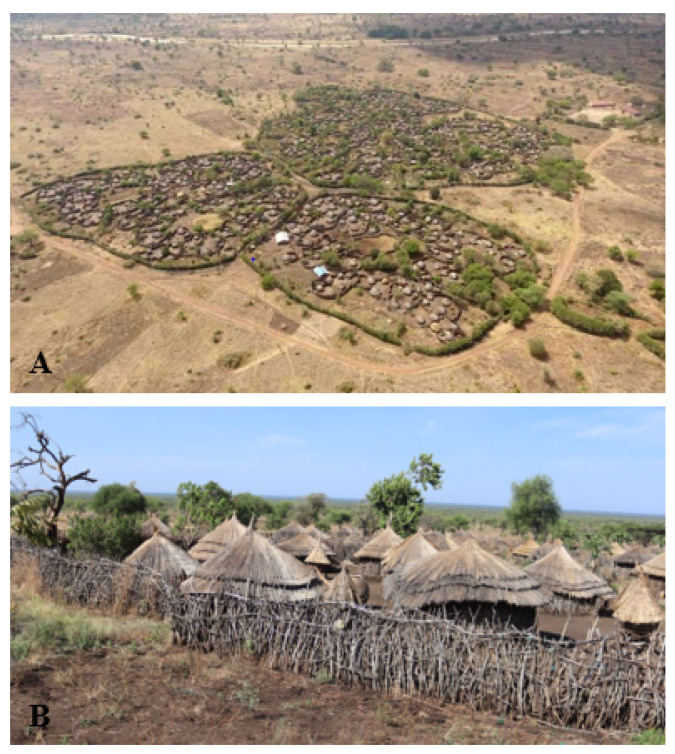
Aerial view of *manyatas* in the Nagule Angolol parish (**A**). Each of the *manyatas* has a perimeter hedge and internal partitions made of stick aggregates, which delineate homesteads. (**B**): A ground view of the organisation of a typical *manyata* in the study area. Boundaries of homesteads and households are demarcated by fences made of sticks.

This study targets the population living in three out of five parishes in the Ngoleriet sub-county, Napak district, Northeastern Uganda (Figure 1). The Napak district is one of the seven districts constituting the Karamoja sub-region, which is considered to be the least socially and economically developed region in Uganda [60,61]. The three study parishes consist of 17 villages, 7 of which are located in Naitakwae parish, 4 in Nagule Angolol parish, and 6 in Nawaikorot parish. The inhabitants of the study area live a semi-nomadic lifestyle, and the majority live in settlements (known as *manyatas*) constituted by groups of a variable number of houses made of sticks and mud, enclosed by a fence. The 17 villages are constituted of a total of 52 *manyattas*, of which 23 are in Naitakwae, 17 in Nawaikorot and 12 in Nagule Angolol. Within the *manyatas*, compounds of individual households or homesteads are usually separated by partitions made of aggregates of sticks (Figure 2A,B). Several *manyatas* constitute a village (Local Council One or LC1), which is administered by the Chairperson LC 1, as per Uganda’s political structure.

The Ngoleriet sub-county is about 10 km away from the District Health Offices, which are located about 340 km away by road in the northeast of Kampala towards Moroto. Within the sub-county, there are two health facilities: Ngoleriet Health Centre (HC) II and Kangole HC III, nine primary schools and three secondary schools. Health Centre II and HC III are headed by enrolled nurses and clinical officers, respectively, and they offer free medical services, as per the structure of Uganda’s healthcare system. In 2019, the District Health Office reported that the residents of Napak district were highly affected by tungiasis. Francis Mutebi and George Mukone undertook a fact-finding visit to Napak to verify the occurrence of tungiasis in areas considered to be highly endemic. A rapid assessment for tungiasis [62] in 11 villages located in the parishes of Naitakwae, Nagule Angolol and Nawaikorot in November 2020 revealed that of the 666 individuals examined, 456 (68.5%) had tungiasis (unpublished data). Moreover, many patients had co-morbidities, amongst which were scabies and podoconiosis. None of the affected persons had ever sought medical attention. Medical care was generally inaccessible for the local population as public medical facilities were located far away from most of the villages, were understaffed and lacked medical supplies. No treatments for tungiasis and scabies were available. Moreover, healthcare workers in the few medical facilities lacked the basic knowledge and skills to manage tungiasis. For example, some healthcare workers recommended the application of kerosene to kill embedded sand fleas, a practice which is highly hazardous (unpublished data). 

The living conditions in the study area are generally unhygienic, whereby most compounds were littered with organic matter such as plant residues and household refuse; the floors of houses were dusty and dirty, and most people practised open air defaecation due to a lack of latrines. None of the households had access to electricity. The inhabitants of the Napak district are mainly semi-nomadic pastoralists but also practice subsistence crop farming. Sorghum, maize and sunflower are the major crops grown, while cattle, goats and sheep are the major livestock species reared, majorly by herding. Other animal species, such as donkeys and pigs, are reared in the area in very small numbers. Livestock herding is mainly the responsibility of teenage boys. Routinely, livestock are kept together at night in communal enclosures (*kraals*) in the centre of the *manyatas* for security reasons. During the study period, cattle rustling was rampant, which forced most animal owners (and their herdsmen) to drive livestock to guarded communal grounds far away from human settlements. However, a few households kept some animals near their houses. The burning of charcoal and bee-keeping are other important economic activities in the area. Crop farming and the marketing of household produce are primarily the responsibility of teenage girls and their mothers. 

### 2.2. Study Design

This cross-sectional survey marks the initiation of a humanitarian project aimed at reducing tungiasis in the population of the three target parishes. The humanitarian project involved repeated rounds of human and animal treatment against tungiasis with dimeticone (Nyda^®^), combined with the public health education of the communities, emphasising body hygiene and environmental sanitation. Initially, we enumerated all households and inhabitants in the three study parishes constituted by 17 villages during a census from 14 December to 17 December 2020. Subsequently, we conducted a survey of tungiasis from 1 February to 18 March 2021, which was a dry season, a period characterised by high prevalence and transmission rates for tungiasis [3,63]. This survey provided the baseline data on the status of tungiasis at the start of the One Health control project.

All permanent residents in the study area were included. Permanent residents were defined as individuals having a home in a *manyata* located in the study area where they had stayed for at least four days per week for the last three months. Other individuals present were classified as visitors. For household members who were absent at the first visit, the household was visited again at least twice before they were considered absent. A household was defined as a group of people who prepare and eat meals together but do not necessarily live in the same house. On enrolment, all participants were allocated unique identification numbers to ensure consistency and anonymity. At the household level, census information was provided by the household head (the overall leader of the family) or the household caretaker (the individual who takes care of the home affairs).

### 2.3. Data Collection

In both the census and the survey, house-to-house visits were made, and data were mainly collected through questionnaire interviews, direct observation, and physical examination of all humans and household animals. Forms designed with the Open Data Kit (ODK collect) were used to record the data. The forms were downloaded onto mobile phones for field-based data collection. Census data included the demographic information of all household members, animal husbandry practices and the socioeconomic characteristics of household members. The households were mapped using GPS. During the baseline examination, two different sets of data collection forms were used: one for individual household member tungiasis examinations and another for animal tungiasis screening. From humans, data were collected on identity, the occurrence of tungiasis and the intensity of sand fleas, plus other skin co-morbidities. Six species of domestic mammals (cats, dogs, cattle, sheep, pigs and goats) were examined for tungiasis. Animal data included animal characteristics such as species, age, sex and breed, as well as the presence of tungiasis and other ectoparasites. 

The data sets were collected during periods of the day when most household members were expected to be at home, i.e., in the afternoon (between 1 p.m. and 5 p.m.) during normal working days, when the majority were home from work, and between 9 a.m. and 2 p.m. on weekends. To minimise the stigmatisation of people affected by tungiasis, all interviews and examinations were carried out in a private location selected by the individual.

All field activities were implemented by eight village tungiasis health workers (VTHWs) per parish, who were specially recruited and trained for the study. The training of VTHWs mainly focused on ethical issues during data collection, the physical examination for tungiasis in humans and animals, questionnaire surveys, data entry and the treatment of affected persons and animals. Each group was supervised by a social worker or a project nurse. All the 24 VTHWs were residents of the Ngoleriet sub-county who were fluent in the local language Ngakarimojong and English. The VTHWs were supported by state-established village health teams (VHTs) (we had initially planned to work with VHTs to collect data, but this was not feasible as many of them could not operate the mobile data collection devices and were not fluent in English, which was necessary for data documentation) and the Local Council One (LC1) leaders, who participated as mobilisers and field guides. 

Hands and legs were carefully examined for tungiasis, and individuals were asked if they had sand flea lesions on other body parts. For those who reported tungiasis lesions on other body parts and were willing to be examined, the entire body was examined. In animals, the entire body was examined for tungiasis. Before the examination of humans and animals for tungiasis, the feet or digits were washed with a brush, soap and water to remove any organic matter that could obscure the visualisation of embedded sand fleas. Diagnosis of tungiasis was based on the morphology of embedded sand fleas, as described by Eisele et al. [21]. Embedded sand fleas, which presented as dark brown to black spots in the centre of a hyperemic rim or yellow-to-white nodular lesions of 2–12 mm in diameter, were categorised as viable. Raised circular brown to black patches or shallow circular skin craters with necrotic edges were characterised as avital lesions. Skin sores from which embedded sand fleas had been completely or partially removed were categorised as manipulated lesions. 

### 2.4. Data Management and Statistical Analysis

Completed data forms were uploaded onto a web-based data repository within one day. Before statistical analysis, the data sets were cleaned and validated in Microsoft Excel. Statistical analysis was performed using Microsoft Excel (2016), R statistical package (version 4.1.2, R Core team 2021) and Stata 15 (Statacorp LLC, 2017, 4905 Lakeway Drive College Station, Texas 77845-4512, USA). The 95% confidence intervals of proportions were calculated using Stata 15. The chi-square test was used to compare the prevalences of tungiasis among groups such as genders, age groups or animal species. The age of infected and uninfected people was compared using a t-test (mean comparison test). Spearman’s rank correlation coefficient was used to assess the relationship between the prevalence of tungiasis in animals and humans at the village level, the intensity of ectopic site infections and total intensity among humans, as well as the prevalence of tungiasis and the median number of lesions among humans at the village level. The Wilcoxon rank-sum test was used to compare differences in sand flea numbers (intensity of tungiasis) between two groups, e.g., the intensity of lesions among villages, genders, animal species and age groups. P-values below 0.05 were considered significant. The intensity of tungiasis was defined as the number of lesions present, irrespective of whether they were manipulated, live or dead. Intensities of embedded sand fleas were defined as mild, moderate and (severe) heavy infections when they had 1–5, 6–30 and above 30 lesions, respectively [24]. 

## 3. Results

### 3.1. Census and Characteristics of the Study Population 

A total of 1334 households were identified in the three parishes of Nagule Angolol (*n* = 380; 28.5%), Naitakwae (*n* = 457; 34.3%) and Nawaikorot (*n* = 497; 37.3%). Household sizes in the study area were highly variable (mean = 4 people, range = 1–15). In the three parishes, 5482 people were identified (Table S1); 3273 (59.7%) were female and 2209 (40.3%) were male. 

Out of the 5482 residents, 4035 (73.6%) were available and consented to participate in the baseline examination (Table S1). The participating residents belonged to 1278 households (Table S2). Seven residents (0.1%) declined to participate in the study. In addition to residents, 13 visitors were examined. Other residents were not traceable during the baseline examination period. The majority were men who had relocated their animals to other areas for safety following the upsurge of animal raids. The mean age of household heads was 49 years (range = 12–115). Most households were headed by females (*n* = 770, 60.3%), and the majority of household heads (*n* = 1097, 85.8%) had not attained any form of formal education (Table S2). Most household heads were Christians (*n* = 1213, 95%). Disabilities (mental or physical) among household heads were common (*n* = 286, 22.4%).

Among the examined children, one-third attended school (*n* = 789; 33.9%). Most adults did not have any formal education (*n* = 1436; 84.3%), while 10.3% (*n* = 176) had started but never completed primary education, and only 2% (*n* = 34) had completed primary school as their highest level of education. Only 3.4% (*n* = 58) had studied beyond the primary level. Hunger was a common problem in the area. In most households, people only had one meal per day (*n* = 872; 73.3%), and in 6.9% of households (*n* = 88), food was so scarce that they had not had any meal the previous day (Table S3). Defaecation in the bush was the main method of faeces disposal among households in the community (*n* = 966, 75.6%).

Out of the 1278 households that participated in the study, 568 (44.4%) were keeping animals (Table S4), although the number of animals kept on the compound or near homesteads was small. Cattle (N = 4899), sheep (N = 5716), goats (N = 4162) and pigs (N = 34) were the major livestock. Dogs (N = 300) and cats (N = 390) were the major companion animals in households, although donkeys were also present. Chickens, ducks, turkeys, guinea fowls and pigeons were the poultry species kept in homes. Only three (0.5%) households had pigs, of which two controlled ectoparasites using commercial ectoparasiticides. 

There were 134 dog-owning households, of which the majority allowed their dogs to roam freely (*n* = 132; 98.5%). Only 15 dog-owning households controlled ectoparasites. There were 238 cat-owning households, of which the majority allowed their cats to roam freely (*n* = 237; 99.6%). Only 37 (15.6%) cat-owning households controlled ectoparasites in their cats. Poultry was found in 18.2% of households (*n* = 232). Rodents were reported to be present on the compound by 96% (*n* = 1227) of the participating households.

### 3.2. Prevalence of Tungiasis in Households and Residents

**Figure 3:**
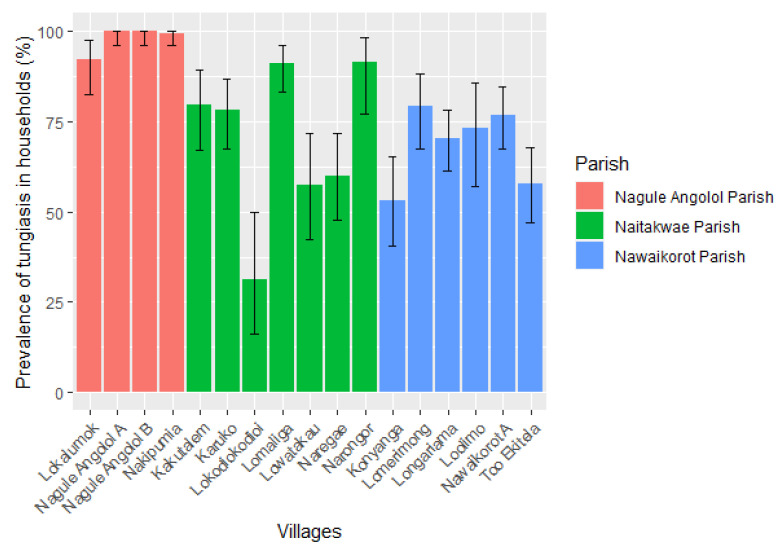
Prevalence of human tungiasis among households in the 17 study villages. The error bars show a 95% confidence interval (*n* = 1278).

Individuals from 1278 households were physically examined, and in 78.7% (*n* = 1006), one or more individuals had tungiasis (Figure S1A). The Nagule Angolol parish had the highest proportion of households with tungiasis infections (98.4%). In the Naitakwae and Nawaikorot parishes, the proportions of affected households were 73.2% and 68.3%, respectively. In two villages of the Nagule Angolol parish, tungiasis infections were present in all households (Figure 3). 

Out of the 4035 examined individuals, 2534 (62.8%) had at least one sand flea lesion in their body (Table 1). The Nagule Angolol parish had the highest prevalence of human tungiasis (84.6%), followed by Naitakwae (49.3%) and Nawaikorot (47.8%). The prevalence of human tungiasis in the 17 villages varied significantly (χ^2^ = 755.70, *p* < 0.001), from 11.5% in the Lokodiokodioi village in the Naitakwae parish to 91.9% in Nagule Angolol A in the Nagule Angolol parish (Table 1). The proportion of infected males (970/1480; 65.5%) was slightly but significantly higher than that of females (1564/2555; 61.2%), χ^2^ = 7.51, *p* = 0.01. Among children, boys were also more affected (67%) than girls (60.4%), χ^2^ = 10.88, *p* = 0.001.

**Figure 4:**
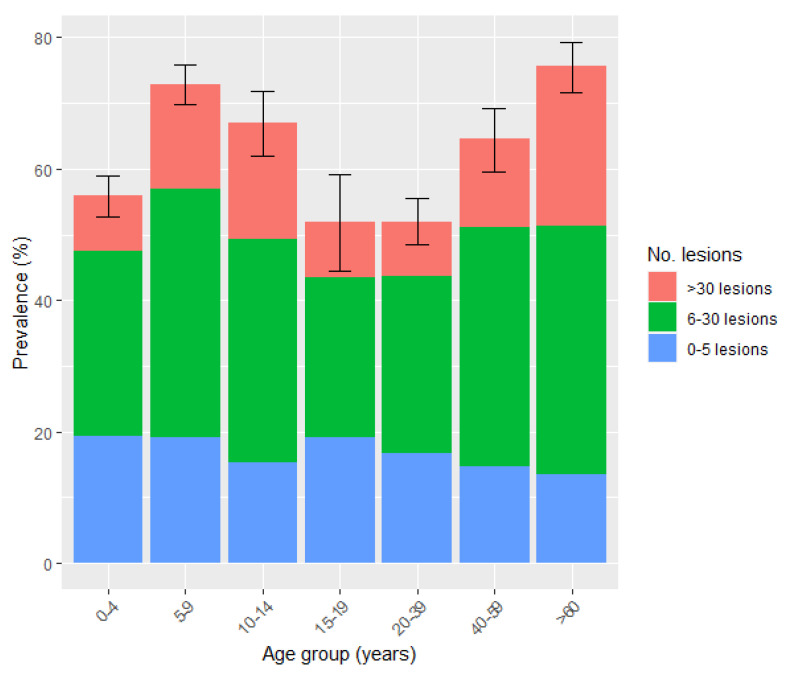
Age-specific prevalence and intensity of human tungiasis. Error bars represent a 95% confidence interval (*n* = 4035).

There was no significant difference in the prevalence of tungiasis between adults (*n* = 1052; 61.7%) and children/adolescents (*n* = 1482; 63.6%), χ^2^ = 1.43, *p* = 0.23. Additionally, the age of infected individuals (mean age = 23.9 years, range < 1–102) did not differ significantly from that of individuals who were not infected (mean age = 20.5 years, range ≤ 1–115), t = −4.38, *p* = 1.00. Age-specific prevalence followed an S-shaped curve, with the highest prevalence occurring in children of 5 to 9 years and the elderly (Figure 4). 

### 3.3. Intensity of Human Infections

**Figure 5:**
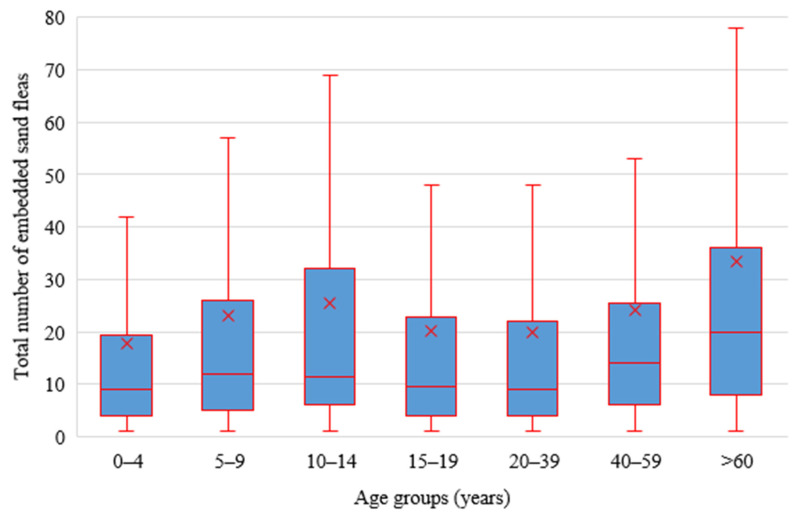
Intensity of infection according to age groups (x represents the mean intensity for each of the age groups, outliers have been excluded from the plot).

The median number of sand flea lesions (intensity) among affected residents was 11 (IQR = 5–26). The majority of affected individuals had moderate infections (51.5%, *n* = 1304), followed by mild infections (*n* = 696; 27.5%) and heavy (severe) infections (*n* = 534; 21%); see Figure 5. Among the severe infections, 96 individuals (3.8% of those infected) had more than 100 lesions (median = 141; IQR = 116–184). These 3.8% carried 26.6% (15,648/58,790) of the sand flea load among humans. Of the 96 individuals, the majority (*n* = 75; 78%) were from the Nagule Angolol parish and were female (*n* = 58; 60.4%).

The intensity of tungiasis between infected males (median = 11; IQR = 5–24) and females (median = 12; IQR = 5–27) did not differ significantly (U = 1.31, *p* = 0.19). However, among children, boys had slightly higher infection intensities (median = 11, IQR = 5–26) than girls (median = 10; IQR = 4–24), U = −2.03, *p* = 0.04. The intensity of tungiasis among adults (median = 13; IQR = 5–28) was slightly but significantly higher than in children (median = 10; IQR = 5–25); U = 2.90, *p* = 0.004. The highest intensity of infection was observed in the elderly (>60 years) (median = 20; IQR = 8–36), children of 5 to 9 years (median = 12; IQR = 5–26) and children of 10 to 14 years (median = 11.5; IQR = 6–32), while children of >1 year to 4 years had the lowest intensity (median = 9; IQR = 4–20) (Figure 5). 

**Figure 6:**
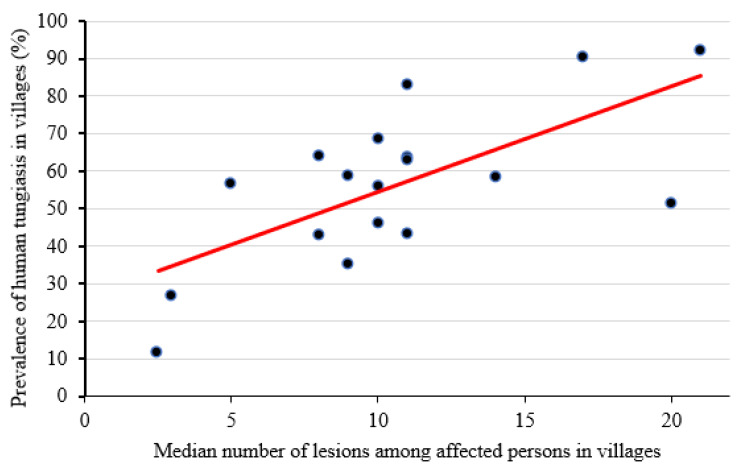
The prevalence of human tungiasis in villages and the median number of lesions were positively correlated at the village level (rho = 0.58, *p* = 0.01).

There was a strong positive correlation between the prevalence of tungiasis and the median number of tungiasis lesions among infected persons at the village level (rho = 0.58, *p* = 0.01), Figure 6. The majority of the tungiasis lesions (50.1%) were manipulated. Ectopic lesions (on other sites apart from the feet) were located on elbows, ankles, fingers, knees and palms of hands and constituted only 1.9% (*n* = 1113) of the total number of lesions detected (*n* = 58,799). Of the 2534 affected individuals, 6.4% (*n* = 162) had ectopic lesions. The intensity of infection of the ectopic sites showed a strong positive correlation with the total infection intensity (rho = 0.68, *p* < 0.001). 

### 3.4. Occurrence of Other Skin Pathology in Humans

A total of 1662 (41.2%) study participants had at least one other skin condition apart from tungiasis. Among other skin pathological skin conditions, the commonest was scabies (*n* = 1625; 40.3%). There were also cases of fungal infections (*n* = 70; 1.7%), head lice (*n* = 28; 0.7%), cutaneous larva migrans (*n* = 17; 0.4%), septic wounds other than those caused by the extraction of *T. penetrans* (*n* = 6; 0.15%), podoconiosis (*n* = 27; 0.67%), warts (*n* = 1; 0.03%,) and unclassified skin rashes (*n* = 2; 0.05%). Fifty percent (*n* = 1270) of individuals with tungiasis had another type of skin pathology. Forty-nine percent (*n* = 1239) of individuals with tungiasis also had scabies. Scabies occurred in 75% (*n* = 72) of the most severe cases (over 100 lesions) of tungiasis. Of the 17 cases of CLM, 12 (70.6%) occurred as a co-morbidity with tungiasis. 

### 3.5. Tungiasis among Visitors

A total of 13 visitors (see Material and Methods section) were examined, of which 11 were children and 2 were adults. All adults, as well as 8 of the 11 children, were infected by *T. penetrans* (10/13 visitors; 76.9%). The median number of embedded sand fleas among the visitors was 23 (range = 2–87). As was the case with residents, most of the lesions among visitors were manipulated (134/239; 56.1%). 

### 3.6. Prevalence and Intensity of Animal Tungiasis

Of the total of 15,501 domestic mammals (dogs, cats, sheep, goats, pigs and cattle) reported by the respondents in the study area, 395 (2.5%) were examined for tungiasis. The majority of the animals had been relocated to distant locations due to insecurity (animal raiding). Examination of animals was performed either from household premises (22%, *n* = 87) or from communal night bomas (community kraals) within a *manyatta* (78%, *n* = 373). The 395 animals belonged to 136 households (23.9%) out of the 568 animal-owning households enrolled during the baseline examination. Nineteen percent (*n* = 26) of the households had at least one infected animal (Figure S1B). The overall prevalence of animal tungiasis was 14.2% (*n* = 56). Tungiasis was detected among 80% of pigs (8/10; CI 44.4–97.5), 24% of dogs (22/92; CI 15.6–33.9), 16% of goats (14/86; CI 9.2–25.8), 8% of cats (7/86; CI 3.3–16.1) and 5% of sheep (5/102; CI 1.6–11.1). The prevalence of tungiasis among animals examined at the community kraal level (*n* = 17; 19.5%) did not differ significantly from that of animals found within household premises (n = 39; 11.9%), χ^2^ = 3.45, *p* = 0.06. At the parish level, the prevalence of animal tungiasis was 23.8% (31/130) in Nawaikorot, 15.5% (18/116) in Nagule Angolol and 4.7% (7/149) in Naitakwae. The prevalence of animal and human tungiasis at the village level was positively correlated (rho = 0.28), but the correlation was not significant (*p* = 0.3). 

The median number of lesions in affected animals was 3 (IQR = 2–6). Pigs were not only the most affected by tungiasis but also had the highest infection intensity (median = 9.5; IQR 3–23.5) of all infected animal species. The infection intensities of tungiasis-affected pigs differed significantly from those of dogs (median = 3.5; IQR = 2–6; U = −2.22, *p* = 0.03), goats (median = 2; IQR = 1–4, U = -2.69, *p* = 0.01) and sheep (median = 3; IQR = 2–3; U = 2.11, *p* = 0.04), but the difference was not significant from those of cats (median = 2; IQR = 2–8, U = −1.98, *p* = 0.05). The intensity of embedded sand fleas did not differ significantly between animals kept in households (median = 3; IQR = 2–7) and those kept in community kraals (median = 3; IQR = 2–4), U = 1.53, *p* = 0.13. Out of the total 59,120 tungiasis lesions found on infected hosts, animals carried only 0.6% (*n* = 330).

## 4. Discussion

The study presents a comprehensive population-based assessment of tungiasis in Uganda. The overall prevalence of human tungiasis was very high (62.8%) but highly variable (11.5%-92%) among the 17 villages. A heterogeneous distribution of tungiasis has been reported in other endemic areas [2,6]. The extremely high prevalence documented in the general population is comparable to prevalences of 62.1%, reported in children in Kwale, Kenya [27], and 58.7% in children in Wensho, Ethiopia [34]. As a rule, children are the most vulnerable group, carrying the highest burden in endemic communities [24,29]. To the best of our knowledge, the overall prevalence of 62.8% has never been reported before. For example, in Busoga, southeastern Uganda, much lower prevalences of 22.5% and 14.4% were reported from the Mayuge district [40] and animal-keeping households in Bugiri [17], respectively. In other highly endemic regions of sub-Saharan Africa, lower prevalences have been reported in Kenya (57%), Nigeria (45.2%), Cameroon (53%) and Tanzania (42.5%) [5,30,31,33,39,64]. Additionally, lower prevalences of tungiasis were recorded in Brazil (up to 54.8%) [16,18,24,63,65] and Trinidad (up to 31.4%) [42,66].

The extremely high prevalence of tungiasis underscores the public health significance of tungiasis in the study area. The Karamoja sub-region, to which the Napak district belongs, is among the least socially and economically developed parts of Uganda [61]. In most communities, the living conditions are precarious, and access to public health services is very limited. The majority of houses in the study area had earthen floors, and sanitary practices, such as household waste disposal methods, are very poor. Such factors prime the occurrence, persistence and propagation of off-host stages of sand fleas [6]. The situation is exacerbated by prolonged drought periods, which are not only associated with dusty and warm environments, favouring the survival of off-host stages [19,63], but also contribute to water scarcity in the area. Indeed, climate analysis of Karamoja over 35 years (1981–2015) shows that the average temperature has been steadily rising [67]. Water scarcity not only compromises the hygienic practices of the communities [6,29], which is a risk factor for tungiasis, but also negatively impacts subsistence crop and animal agriculture, the major cornerstone for the communities’ livelihoods in Karamoja [67]. This keeps the population trapped in poverty. Poverty primes the existence of various predisposing factors to tungiasis [27,29,34,35,38,40,64,68]. Affected communities cannot afford solid floors, soap, water reservoirs and other basic infrastructure for good body hygiene and environmental sanitation.

Tungiasis-associated morbidity has many negative psychosocial and educational outcomes that impede the social–economic progression of affected individuals, families and, therefore, communities [69]. For example, stigma contributes to social exclusion, while itching, pain, sleeping disturbances, lack of concentration and body deformities affect economic productivity among adults and poor schooling outcomes among school-going children [52,69]. 

In the villages of Nagule Angolol A and Nagule Angolol B, in all households, at least one case of tungiasis occurred (100% household prevalence), and the individual prevalence of human tungiasis was 91.9% and 90.5%, respectively. When compared with other parishes, the Nagule Angolol parish, with its four villages, had the highest prevalence of human tungiasis in the study area (84.6%). The residents of Nagule Angolol live in rather big *manyatas,* which are aggregated in one locality (see Figure 1). In this setting, many households, and, therefore, also people, are concentrated in a limited area, which, in turn, favours the transmission of sand fleas between members of various households. Amongst others, gregariousness is a coping mechanism for security threats, but it concentrates pathogens due to crowding. Two studies in Kenya have identified a high density of people per sleeping room as a risk factor for tungiasis [6,70]. 

Among the different age groups, the prevalence of human tungiasis followed a typical S-shaped curve trend, where children of 5–9 years and the elderly (≥60 years) had the highest prevalence, as reported before in other studies [24,27,29,34,35,65]. The S-shaped curve of age-specific prevalence has been attributed to variations in behavioural and hygienic practices in children and the sedentary way of life and neglect of elderly persons in resource-poor settings [6,24,65]. 

A finding hitherto not described is that age groups of 0–4, 15–19 and 20–39 years had prevalences of 50% and above. Very young children usually receive maximum care from parents and have little contact with the soil, the source of infection with sand fleas. On the other hand, teenagers and younger adults are usually considered less likely than young children to engage in risky behaviours, such as playing in dusty places, and they often take good care of their body hygiene. In the study population, children and adolescents of 10–19 years of age are usually away from home most of the time during the day, either herding animals in the case of boys or trading in the case of girls. This suggests that family members become infected by *T. penetrans* at night when they are sleeping indoors, irrespective of their age and hours spent around the homesteads. An analysis of soil samples from indoors showed that in almost all samples, the off-host stages of *T. penetrans* were present (F. Mutebi, unpublished observation 2022).

Males had a slightly higher prevalence of tungiasis (65.5%) than females (61.2%). This finding conforms with most other studies that have reported higher human tungiasis prevalence among males than females [6,24,29,38,42,65,66]. However, there are variations in the effect of gender on the prevalence of tungiasis infections among communities. A study from Nigeria reported no significant gender difference in prevalence [41], and in a study in Brazil, more females than males were affected [25]. The variations in prevalence between the two genders have been mainly attributed to differences in adherence to proper hygienic practices, with girls typically being more concerned about their personal body hygiene than boys [29]. On the other hand, the intensity (the number of sand-flea-associated lesions) of tungiasis in the study area did not differ between genders, except among children, as boys had more sand flea lesions than girls. Most studies in other endemic communities reported higher tungiasis intensities among males compared to females. [24,25,65]. However, a study in Uganda among animal-keeping households reported no significant difference in infection intensity among boys and girls [17]. 

An astoundingly high proportion of individuals with human tungiasis (72.5%) had moderate or heavy infections (≥six lesions), which indicates intense transmission. This deviates from the known pattern of distribution of *T. penetrans* lesions among infected hosts, where the majority of lesions are carried by a few hosts [4,17,24]. A strong correlation between prevalence and intensity has been described before [4,5,6,17]. Thus, we can assume that the very high prevalence of tungiasis in the study population contributes to the high intensity of tungiasis infection among affected persons and vice versa. Accordingly, the pattern of the age-specific infection intensity of tungiasis followed a similar trend to age-specific prevalence. This finding differs from the U-shaped distribution of infection intensities in age groups described in Balbino, Brazil [24]. 

Although their number was small, the majority (92%) of the 13 visitors found within the study area were infected, and moreover, with high infection intensities of tungiasis (median = 23). This demonstrates that visitors or incomers from other locations are potential sources of new infections in the community. Thus, all efforts aimed at eliminating tungiasis within the community should also target visitors and encourage new entrants into the area to report to medical facilities for immediate treatment if infected.

Tungiasis is associated with considerable impairment in the quality of life [26], and no medicine was available in the study area. Thus, it is not surprising that half of the total number of lesions in humans were manipulated with sharp instruments, such as pins and thorns. Similar observations have been made in Brazil and Madagascar [24,65,71]. The high rate of manipulation of embedded sand fleas suggests an attempt to eliminate or minimise the morbidity attributable to tungiasis despite the pain that is associated with the manipulation of lesions and the risk of secondary bacterial infections. Due to the inaccessibility of effective alternative treatments for tungiasis in many endemic communities, the mechanical extraction of sand fleas remains the most widely used method [59,72]. Apart from pain, the practice of mechanically removing embedded sand fleas from the body has many limitations. Firstly, it is not practicable in environments with high transmission rates, where individuals carry a high number of embedded sand fleas. Secondly, unsterile extractions and the contamination of extraction sores by soil from bare-footed individuals may introduce secondary bacterial infections, including tetanus, which is potentially fatal [48,53]. In addition, the sharing of extraction objects, which is a common practice in poor communities [55], may contribute to the transmission of blood-borne pathogens, including HIV and hepatitis B viruses [2]. Currently, an effective tungicidal medical device, dimeticone (Nyda^®^), exists [73,74]. Nyda^®^ is made of two silicone oils (dimeticones) with proven efficacy against tungiasis [73,74]. It has a physical mode of action, which involves the penetration of flea tissues with a disruption of vital functions, especially respiration, to kill embedded fleas [75,76]. We can expect that availing dimeticone to the endemic communities will facilitate the abandonment of hazardous manual extractions.

Although designed as a population-based study, the coverage of the population was relatively low (73.6% of the population). The majority of residents have a semi-nomadic lifestyle and keep moving in and out of the area in search of food for themselves and their animals, especially during dry seasons. As the study was also undertaken during a particularly long dry season, when many men were herding animals outside the study area, many more females (60%) than males (40%) were present in the villages. During the study period, cattle rustling was also rampant in the area; thus, many men and teenage boys were forced to drive cattle away to more secure places. In some villages, particularly in Konyanga and Kakutalem, many residents were displaced due to animal-rustling-associated insecurity. 

Only a small number of animals (*n* = 395) could be examined. Most animals were kept away from homes due to the escalating animal raids in the area. Although pigs, dogs, cats, sheep and goats were found to be infected, animal tungiasis occurred only in a small proportion of the animals (14.2%). Animals carried only 0.6% of the total number of lesions among infected hosts in the study area. Previously, pigs were identified as the major animal host of tungiasis in Busoga, Uganda [17] and in Nigeria [15]. Indeed, in the current study, a high proportion of pigs (80%) were found to be infected, but the absolute number was small. Thus, in the study area, zoonotic transmissions of tungiasis may be of limited significance in the epidemiology of human tungiasis. Nonetheless, animals infected with *T. penetrans* increase the risk and intensity of human tungiasis [16,17,18]. One Health principles integrating human, animal and environmental interventions should be applied for effective tungiasis control.

## 5. Conclusions

The study demonstrates that zoonotic tungiasis is highly prevalent in the three study parishes of the Napak district. The prevalence and intensity of tungiasis were higher in humans than in domestic animals. Half of the total number of tungiasis lesions identified among humans had been manipulated with sharp objects. Effective control measures based on One Health principles are necessary to decrease the prevalence and intensity of tungiasis and hazardous manual extractions in this setting. 

## Figures and Tables

**Table 1:** Prevalence of human tungiasis in the 17 villages of the three parishes.

Village	Number Examined	Number Infected (n, %)	95% CI
**Naitakwae Parish**			
Kakutalem	165	97 (58.8)	50.9–66.4
Karuko	223	114 (51.1)	44.4–57.9
Naregae	189	81 (42.9)	35.7–50.2
Narongor	124	79 (63.7)	54.6–72.2
Lokodiokodioi	104	12 (11.5)	6.1–19.3
Lomaliga	304	195 (64.1)	58.5–69.5
Lowatakau	138	37 (26.8)	19.6–35.0
**Naitakwae prevalence**	**1247**	**615 (49.3)**	**46.5–52.1**
**Nawaikorot Parish**			
Konyanga	148	68 (46.0)	37.7–54.3
Nawaikorot A	240	136 (56.7)	50.1–63.0
Too Ekitela	210	74 (35.2)	28.8–42.1
Lomerimong	158	92 (58.2)	50.1–66.0
Longariama	347	150 (43.2)	38.0–48.6
Loolimo	91	51 (56.0)	45.3–66.4
**Nawaikorot prevalence**	**1194**	**571 (47.8)**	**45.0–50.7**
**Nagule Angolol Parish**			
Nagule Angolol A	434	399 (91.9)	89.0–94.3
Nagule Angolol B	283	256 (90.5)	86.4–93.6
Nakipumia	629	523 (83.1)	80.0–86.0
Lokalumok	248	170 (68.5)	62.4–74.3
**Nagule Angolol prevalence**	**1594**	**1348 (84.6)**	**82.7–86.3**
**Overall**	**4035**	**2534 (62.8)**	**61.3–64.3**

## Data Availability

The data presented in this study are available on request from the corresponding author.

## Supplementary Materials

The following supporting information can be downloaded at: https://www.mdpi.com/article/10.3390/tropicalmed8020111/s1, Table S1: Census of residents and number examined in the 17 study villages. Table S2: Socio-demographic characteristics of households (N = 1278). Table S3: Economic features, sanitation, hygiene and living conditions in the households (N = 1278). Table S4. Animal-keeping practices in the participating households (*n* = 568). Figure S1: Infection of tungiasis on the human hand (A) and tungiasis infection on the digits of a pig (B).
