# Supplementary material for: Prevalence and Infection Intensity of Human and Animal Tungiasis in Napak District, Karamoja, Northeastern Uganda
Source: Trop Med Infect Dis. 2023 Feb 11;8(2):111. doi: 10.3390/tropicalmed8020111 (PMC9963877; doi:10.3390/tropicalmed8020111)

Figure S1: Infection of tungiasis on the human hand (A) and tungiasis infection on the digits of a pig (B)

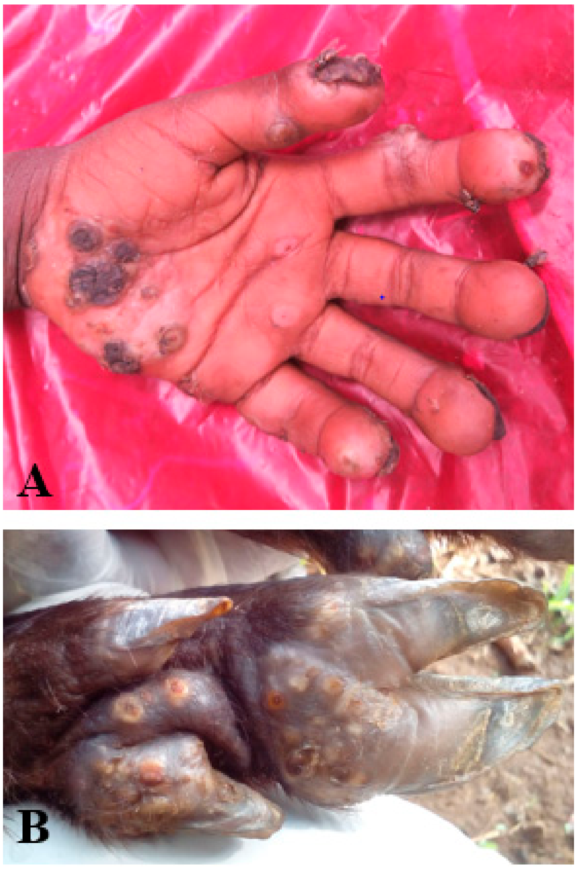

Supplement: Supplementary file 1 [file tropicalmed-08-00111-s001.zip › Figure S1.pdf]
