# Supplementary material for: Prevalence and Infection Intensity of Human and Animal Tungiasis in Napak District, Karamoja, Northeastern Uganda
Source: Trop Med Infect Dis. 2023 Feb 11;8(2):111. doi: 10.3390/tropicalmed8020111 (PMC9963877; doi:10.3390/tropicalmed8020111)
Supplement: Supplementary file 1 [file tropicalmed-08-00111-s001.zip › Table S1.pdf]

Table S1. Census of residents and number examined in the 17 study villages.

Village	Number of people identified (%)	Number examined (%)	Proportion of people present / examined (%)
**Naitakwae parish**			
Naregae	266 (4.8)	189 (4.7)	71.1
Kakutalem	300 (5.5)	165 (4.1)	55.0
Karuko	310 (5.7)	223 (5.5)	71.9
Narongor	187 (3.4)	124 (3.1)	66.3
Lowatakau	197 (3.6)	138 (3.4)	70.1
Lokodiokodioi	148 (2.7)	104 (2.6)	70.3
Lomaliga	534 (9.7)	304 (7.5)	56.9
**Sub total**	**1,942 (35.4)**	**1,247 (30.9)**	**64.2**
**Nawaikorot parish**			
Lomerimongo	189 (3.4)	158 (3.9)	83.6
Longariama	449 (8.2)	347 (8.6)	77.3
Konyanga	220 (4.0)	148 (3.7)	67.3
Loolimo	115 (2.1)	91 (2.3)	79.1
Nawaikorot A	311 (5.7)	240 (6.0)	77.2
Too Ekitela	328 (6.0)	210 (5.2)	64.0
**Sub total**	**1,612 (29.4)**	**1,194 (29.6)**	**74.1**
**Nagule Angolol parish**			
Nagule Angolol A	495 (9.0)	434 (10.8)	87.7
Nagule Angolol B	439 (8.0)	283 (7.0)	64.5
Nakipumia	710 (13.0)	629 (15.5)	88.6
Lokalumok	284 (5.2)	248 (6.1)	87.3
**Sub total**	**1,928 (35.2)**	**1,594 (39.5)**	**82.7**
**Overall Total**	**5,482 (100)**	**4,035 (100)**	**73.6**
