# Supplementary material for: Prevalence and Infection Intensity of Human and Animal Tungiasis in Napak District, Karamoja, Northeastern Uganda
Source: Trop Med Infect Dis. 2023 Feb 11;8(2):111. doi: 10.3390/tropicalmed8020111 (PMC9963877; doi:10.3390/tropicalmed8020111)
Supplement: Supplementary file 1 [file tropicalmed-08-00111-s001.zip › Table S2.pdf]

Table S2: Socio-demographic characteristics of households (N=1,278).

Characteristic	Categories	Frequency (%)
Marital status of household head	Polygamous marriage	658 (51.5)
	Monogamous marriage	430 (33.6)
	Widowed	145 (11.3)
	Single	34 (2.7)
	Divorced/Separated	11 (0.8)
Gender of household head	Female	770 (60.3)
	Male	508 (39.7)
Education level of household head	None[1]	1,097 (85.8)
	Some Primary[2]	122 (9.5)
	Completed primary	19 (1.5)
	Some secondary[2]	29 (2.3)
	Completed secondary and beyond	11 (0.9)
Religion of household head	Christian	1,213 (94.9)
	Traditional religion	54 (4.2)
	Muslim	6 (0.5)
	None	5 (0.4)
Ethnicity of household head	Karamojong	1,275 (99.8)
	Iteso	2 (0.1)
	Others (Kenyan origin)	1 (0.1)
Disability of household head	None	992 (77.6)
	Physical	266 (20.8)
	Mental	15 (1.2)
	Both physical and mental	5 (0.4)

[1]Has never been to any formal school.
[2]Attended formal primary or secondary school, respectively but never completed it.
