# Supplementary material for: Prevalence and Infection Intensity of Human and Animal Tungiasis in Napak District, Karamoja, Northeastern Uganda
Source: Trop Med Infect Dis. 2023 Feb 11;8(2):111. doi: 10.3390/tropicalmed8020111 (PMC9963877; doi:10.3390/tropicalmed8020111)
Supplement: Supplementary file 1 [file tropicalmed-08-00111-s001.zip › Table S3.pdf]

**Table S3:** Economic features, sanitation, hygiene and living conditions in the households (N=1,278).

| Characteristics | Categories | Frequencies (%) |
|---|---|---|
| Occupation of household head | Peasant/Pastoralist | 235 (18.4) |
| | Casual laborer | 267 (20.9) |
| | None | 657 (51.4) |
| | Small business | 100 (7.8) |
| | Formal job sector | 17 (1.3) |
| | Others (VHT and LC leader) | 2 (0.2) |
| Ownership of house | Own house | 803 (62.8) |
| | Rented house | 22 (1.7) |
| | Temporarily accommodated (squatter) | 453 (35.5) |
| Possessing own farming land | Yes | 1, 151 (90.1) |
| | No | 127 (9.9) |
| Number of meals per day | Irregular[1] | 88 (6.9) |
| | 1 | 872 (73.3) |
| | 2 | 296 (24.9) |
| | 3-4 | 21 (1.6) |
| Construction of house walls | Mud and wattle | 1,241 (97.1) |
| | Blocks/bricks and mud | 13 (1.0) |
| | Bricks and sand/plastered | 24 (1.9) |
| Construction of the roof | Natural/grass | 1,201 (94.0%) |
| | Iron sheets | 38 (3.0%) |
| | Tiles | 39 (3.0%) |
| Floor type | Concrete/tiles/terrazzo | 6 (0.5) |
| | Loose soil/dusty/sandy | 400 (31.3) |
| | Compact soil/smeared with cattle dung | 871 (68.2) |
| Source of water | Shared community borehole | 1,170 (91.5) |
| | Shared community tap | 93 (7.3) |
| | Tap in compound | 12 (0.9) |
| | Rain water/river | 3 (0.3) |
| Disposal of faeces | Bush | 966 (75.6) |
| | Traditional latrine | 292 (22.9) |
| | Ventilated improved pit latrine | 18 (1.4) |
| | Flush toilet | 2 (0.1) |
| Disposal of household waste | Burning | 404 (31.6) |
| | Garden/bush near compound | 402 (31.5) |
| | Garden/bush far from compound | 279 (21.8) |
| | Pit | 192 (15.0) |
| | Animal dwellings | 1 (0.1) |
| Use of soap when cleaning the body | Yes | 568 (44.4) |
| | No | 710 (55.6) |

[1]Missed a meal a day before and are not always sure of getting what to eat for any day.
