# Supplementary material for: Prevalence and Infection Intensity of Human and Animal Tungiasis in Napak District, Karamoja, Northeastern Uganda
Source: Trop Med Infect Dis. 2023 Feb 11;8(2):111. doi: 10.3390/tropicalmed8020111 (PMC9963877; doi:10.3390/tropicalmed8020111)
Supplement: Supplementary file 1 [file tropicalmed-08-00111-s001.zip › Table S4.pdf]

Table S4. Animal keeping practices in the participating households (n=568).

Animal keeping practice	Category	Frequency (%)
Keeping dogs on compound	Yes	134 (23.6)
Control of ectoparasites in dogs (n=134)	Yes	15 (11.2)
Keeping cats	Yes	238 (41.9)
Control of ectoparasites in cats (n=238)	Yes	37 (15.6)
Keeping goats	Yes	335 (59)
Method of keeping goats (n=335)	Tethering	16 (4.8)
	Herding	319 (95.2)
Keeping sheep	Yes	362 (63.7)
Method of keeping sheep (n=362)	Herding/roaming	358 (98.9)
	Tethering	4 (1.1)
Keeping cattle	Yes	433 (76.2)
Method of keeping cattle (n=433	Herding/roaming	426 (98.4)
	Tethered	7 (1.6)
Keeping donkeys	Yes	7 (1.2)
Keeping chicken	Yes	378 (66.5)
Chicken rearing system (n=378)	Free range	376 (99.5)
	Intensive (deep litter)	2 (0.5)
